# Impact of public transportation use on the mental health of adults: a predictive model of distress, generalized anxiety, and anger

**DOI:** 10.3389/fsoc.2026.1766636

**Published:** 2026-03-17

**Authors:** Esteban Sarmiento-Suarez, Gabriela Rivera-Álvarez, Dulce Bernabel-Tarazona, Diego Valencia-Pecho, Tomás Caycho-Rodríguez, Jonatan Baños-Chaparro

**Affiliations:** 1 Universidad Privada Norbert Wiener, Facultad de Ciencias de la Salud, Programa académico de Psicología, Lima, Peru; 2 Universidad Científica del Sur, Facultad de Psicología, Lima, Peru

**Keywords:** adults, mental health, psychosocial factors, transport, urban mobility

## Abstract

**Introduction:**

Public transport is a daily activity for a large proportion of the urban population and may involve stressful experiences such as overcrowding, delays, or insecurity. These conditions may affect mental health; however, there is still limited evidence exploring its role as a predictor of specific psychological indicators in adults.

**Objective:**

To analyse the perception of public transport experience as a predictor of distress, generalized anxiety, and anger in adults.

**Materials and methods:**

A predictive associative strategy and quantitative approach were employed. A total of 507 Peruvian adults (66.7% women) residing in Metropolitan Lima participated and completed a sociodemographic survey and psychological instruments. Statistical analyses were conducted using structural equation modeling with latent and observed variables.

**Results:**

The hypothetical model showed acceptable fit: CFI = 0.93, RMSEA = 0.05 [90% CI: 0.049, 0.601], and SRMR = 0.04. Specifically, perception of public transport experience was a positive and statistically significant predictor of distress (β = 0.30, *p* = 0.001), generalized anxiety (β = 0.27, *p* = 0.001), and anger (β = 0.47, *p* = 0.001).

**Conclusions:**

The findings indicate that daily mobility, far from being a neutral activity, represents a meaningful component of emotional wellbeing. Recognizing its impact supports the development of more comprehensive public policies and interventions aimed at improving the emotional quality of life of public transport users.

## 1 Introduction

The rapid process of urbanization, which has led to more than half of the world's population residing in cities, constitutes one of the most significant demographic phenomena of the twenty first century and presents substantial challenges for public health, particularly in the field of mental health (Halbreich, 2023; Okkels et al., 2018; Ventriglio et al., 2020). In this context, the accumulation of minor daily hassles—such as constant noise, traffic congestion, time pressure, insecurity, or lack of social cohesion—has been identified as a stronger predictor of psychological distress than isolated major life events (DeLongis et al., 1982; Haight et al., 2023).

Within this landscape, daily commuting emerges as one of the most chronic, harmful, and often unavoidable stressors of contemporary urban life (Tao et al., 2023; Zhang and Ma, 2025). Research on urban transport has evolved notably in recent decades, with early studies focusing on economic and engineering aspects, prioritizing variables such as travel time, cost, and operational efficiency (Goodman et al., 2020; Stankov et al., 2020). Consequently, urban transport is now recognized as an activity that significantly affects the social and environmental determinants of health (Barros dos Santos and Lima, 2024).

Various studies have shown that the time devoted to daily commuting—particularly long commutes—is associated with adverse effects on general wellbeing and physical health (Wu et al., 2021). Prolonged commuting has also been linked to lower life satisfaction and poorer sleep quality, factors that contribute to psychological exhaustion or burnout among users (Deng et al., 2024). Another key concept is time poverty, understood as the perception of not having sufficient free time for work, family responsibilities, and other activities, which can influence stress and subjective wellbeing due to reduced time availability (Carmichael et al., 2024; Giurge et al., 2020).

Public transport, in particular, exposes users to a series of specific environmental stressors that intensify psychological discomfort. Overcrowding, characterized by the invasion of personal space, has been identified as a major stress factor that increases discomfort and emotional tension; environmental factors such as noise, extreme temperatures, and unpleasant odors have also been associated with adverse physiological and emotional responses (Wang and Zacharias, 2020). Unpredictability—manifested in delays and lack of control over the journey—significantly contributes to perceived stress and frustration among users (Evans et al., 2002). Moreover, negative social interactions, including incivility or conflict with other passengers, can aggravate psychological discomfort during travel (Cochran, 2020).

A growing body of research indicates that the perception of public transport experience can increase psychological distress. Factors such as unpredictability of service, overcrowding, and long travel times are consistently associated with higher levels of perceived stress and exhaustion (Chatterjee et al., 2020; Tsoi and Loo, 2023). For instance, a systematic review found that crowding and traffic congestion during travel heighten distress and negatively affect subjective wellbeing, both during the journey and in subsequent performance at work and home (Chatterjee et al., 2020). Recent studies have also confirmed that unpredictability and lack of reliability in public transport are major sources of stress, exacerbating the sense of lack of control and increasing vulnerability to exhaustion (Tsoi and Loo, 2023).

Similarly, the perception of public transport experience may trigger and exacerbate symptoms of generalized anxiety, particularly in individuals with anxiety disorders, who report feeling “trapped” and overwhelmed by system complexity and information overload, which may provoke panic attacks and avoidance behaviors that affect daily functioning (Ratering et al., 2024). Exposure to transport-related noise is also associated with a higher likelihood of anxiety, especially in more severe cases, although evidence remains limited (Lan et al., 2020). Furthermore, insecurity and fear of crime—particularly among women—increase hypervigilance and defensive behaviors during waiting times and travel, reinforcing anxious states and perceptions of vulnerability (Chowdhury and Van Wee, 2020; Kim, 2019).

Anger in transport settings is often triggered by factors such as congestion, reckless behavior of other users, and situations perceived as unfair, which heighten aggressive responses and the risk of accidents (Karimi et al., 2022). Experiences of anger and stress during travel may also spill over into home or work environments, affecting wellbeing, job satisfaction, and family dynamics, particularly when journeys are long or aversive (Bai et al., 2021). The subjective perception of the journey and self-regulation capacity shape the intensity of this spillover, meaning that coping strategies and flexible work arrangements can mitigate the negative impact of transport-related anger on other life domains (Jachimowicz et al., 2020).

Although the literature confirms comorbid relationships between distress, anxiety, and, to a lesser extent, anger, specific evidence regarding anger in public transport contexts remains scarce. Most transport studies have focused on distress and anxiety, reporting that factors such as congestion, unreliability, and environmental conditions can increase psychological discomfort and anxiety among users (Conceição et al., 2023; Hanaee et al., 2025; Lan et al., 2020). However, research jointly examining anger, distress, and anxiety in public transport contexts is highly limited, particularly in low- and middle-income countries seeking to understand the interaction of these three variables in such settings. Therefore, the aim of the study was to analyse the perception of public transport experience as a predictor of distress, generalized anxiety, and anger in adults.

## 2 Materials and methods

### 2.1 Participants

This study was conducted using a predictive associative design with latent variables, adopting a quantitative approach and a cross-sectional scope. Data collection took place between January and April 2025 through an online survey created using the Google Forms platform. The questionnaire was distributed via the researchers' social media accounts and included clear information about the study's objectives, anonymity assurances, academic purpose, data management procedures, and the participants' informed consent. The use of digital surveys provides several methodological advantages, such as broader reach and accessibility for participant recruitment, greater systematic control of responses, the possibility of incorporating different interactive media formats, and faster and more cost-effective administration of data collection (Gosling and Mason, 2015). The sample size calculation was conducted using the Soper calculator, which is specifically designed for structural equation modeling. In particular, for the proposed model comprising 4 latent variables and 22 observed variables, an anticipated effect size of 0.30, statistical power of 0.99, and a significance level (*p*-value) of 0.05 were specified. The recommended minimum sample size was 277 participants.

A total of 507 Peruvian adults residing in Metropolitan Lima participated in the study through a non-probabilistic online sampling strategy (Table 1). The majority were women (66.7%), with an average age of 33 years (*SD* = 12.05; range = 18-60). Regarding employment, 72.4% reported being currently employed, while 27.6% were unemployed. In terms of marital status, most participants identified as single (72%) or married (25%), with smaller proportions being divorced (2.8%) or widowed (0.2%). The vast majority of respondents lived in urban areas (95.9%). Concerning educational attainment, the most common levels reported were incomplete university studies (32.7%), completed university education (26.2%), and completed technical education (12.2%). With respect to public transportation use, the most frequently reported average daily travel time (including round trips) ranged between 1 and 1.9 h (33.7%), followed by less than 1 h (27.6%), 2 to 2.9 h (20.9%), 3 to 3.9 h (10.8%), more than 4 h (5.5%), and “don't remember” (1.4%).

**Table 1:** Sociodemographic characteristics of the participants.

**Characteristic**	***n* (%)**
**Sex**	
Women	338 (66.7%)
Men	169 (33.3%)
Age	33.3 ± 12.05
**Marital status**	
Single	365 (72%)
Married	127 (25%)
Divorced	14 (2.8%)
Widowed	1 (0.2%)
**Employment status**	
Yes	367 (72.4%)
No	140 (27.6%)
**Area of residence**	
Urban	486 (95.9%)
Rural	21 (4.1%)
**Education**	
Incomplete secondary	3 (0.6%)
Completed secondary	43 (8.5%)
Incomplete technical	20 (3.9%)
Completed technical	62 (12.2%)
Incomplete university	166 (32.7%)
Completed university	133 (26.2%)
Incomplete postgraduate	20 (3.9%)
Completed postgraduate	60 (11.8%)
**Travel time**	
Less than 1 h	140 (27.6%)
1-1.9 h	171 (33.7%)
2-2.9 h	106 (20.9%)
3-3.9 h	55 (10.8%)
More than 4 h	28 (5.5%)
Don't remember	7 (1.4%)

### 2.2 Instruments

**Sociodemographic questionnaire**. A brief demographic questionnaire was used to gather information on gender, age, employment status, marital status, area of residence, educational level, and average commute time using public transportation (round trip).

**Questionnaire on the Use of Public Transportation and Wellbeing (QUPTW)**. This nine-item instrument evaluates individuals' perceptions of public transportation and how these relate to their overall wellbeing (Torales et al., 2024). Responses are provided on a five-point Likert scale ranging from 0 (strongly disagree) to 4 (strongly agree). The total possible score varies between 9 and 45, with higher scores reflecting lower levels of wellbeing. In the present study, the scale's psychometric characteristics were analyzed, showing a unidimensional structure [CFI = 0.99, TLI = 0.99, RMSEA = 0.07 (90% CI: 0.055, 0.086), SRMR = 0.04] and satisfactory internal consistency (ω = 0.88).

**Perceived Stress Scale (PSS)**. This 12-item scale assesses the level of perceived stress experienced during the past month and comprises two dimensions: eustress and distress. It includes both positively and negatively worded statements about stressful situations or life events. In this study, only the distress dimension was used to directly assess negative stress. Responses are rated on a Likert scale ranging from 0 (never) to 4 (very often). Total scores range from 0 to 24, with higher scores indicating greater levels of distress. The Peruvian adaptation of the scale was employed, and the present study reported adequate reliability for the distress dimension (ω = 0.86) (Boluarte-Carbajal et al., 2023).

**Generalized Anxiety Disorder-2 (GAD-2)**. This is a brief version that measures generalized anxiety experienced over the past 2 weeks through two items. Responses are given on a four-point scale ranging from 0 (not at all) to 3 (nearly every day). The sum of the two items produces a total score between 0 and 6, with higher scores indicating greater levels of generalized anxiety. In this study, the Peruvian adaptation was used, which demonstrated adequate reliability (ω = 0.93) (Baños-Chaparro, 2022).

**Dimensions of Anger Reactions-5 (DAR-5)**. The DAR-5 is a five-item scale that evaluates experiences of anger over the past 4 weeks. It uses a five-point Likert scale ranging from 1 (none or almost none of the time) to 5 (all or almost all of the time). Total scores range from 5 to 25, with higher scores indicating more frequent experiences of anger. The Peruvian adaptation was used, and this study showed adequate reliability (ω = 0.86) (Caycho-Rodríguez et al., 2020).

### 2.3 Data analysis

Statistical processing was carried out using the open-source software RStudio. In the first phase, a descriptive analysis of the psychological variables was conducted, employing measures of central tendency and dispersion, such as arithmetic mean and standard deviation, as well as the Pearson correlation matrix. For the interpretation of the correlations, effect size was considered and classified into the following categories: small (*r* = 0.10), medium (*r* = 0.20), large (*r* = 0.30), and very large (*r* = 0.40) (Kallogjeri and Piccirillo, 2023).

In the second phase, an inferential regression analysis based on structural equation modeling (SEM) was applied. The SEM included four latent variables: perception of public transport experience (QUPTW items), distress (PSS distress items), generalized anxiety (GAD-2 items), and anger (DAR-5 items), each measured by their respective observed items. This procedure employed latent variables with the aim of estimating standardized regression coefficients, determining their statistical significance, and assessing the model's goodness of fit. Prior to model estimation, multivariate normality was assessed using Mardia's test. The results indicated significant multivariate skewness (b1p = 56.79, *p* < 0.001) and kurtosis (b2p = 620.67, *p* < 0.001), suggesting violation of the assumption of multivariate normality. Accordingly, the robust maximum likelihood estimator (MLR) was employed (Jongerling et al., 2024). Model validation was carried out at two levels. The first corresponded to global evaluation using the comparative fit index (CFI > 0.90), the root mean square error of approximation (RMSEA < 0.08), and the standardized root mean square residual (SRMR < 0.08) (Jordan Muiños, 2021). When these indices did not reach the expected thresholds, modification indices greater than 10 were examined to identify potential covariances between the errors of observed variables. At the second level, local evaluation focused on examining the standardized regression coefficients (β) and verifying their statistical significance (*p* < 0.05). This approach provided both a general and detailed understanding of the model's behavior and the relationships among the included variables.

### 2.4 Ethical considerations

The study adhered to both international and national ethical guidelines in psychological research. Participants provided informed consent prior to participation. The survey was anonymous, voluntary, and data confidentiality was assured (Hilbig et al., 2022). Additionally, the study was reviewed and approved by the ethics committee of the Universidad Privada Norbert Wiener under registration number 0833-2024-CIEIC-UPNW.

## 3 Results

### 3.1 Descriptive analysis

**Figure 1:**
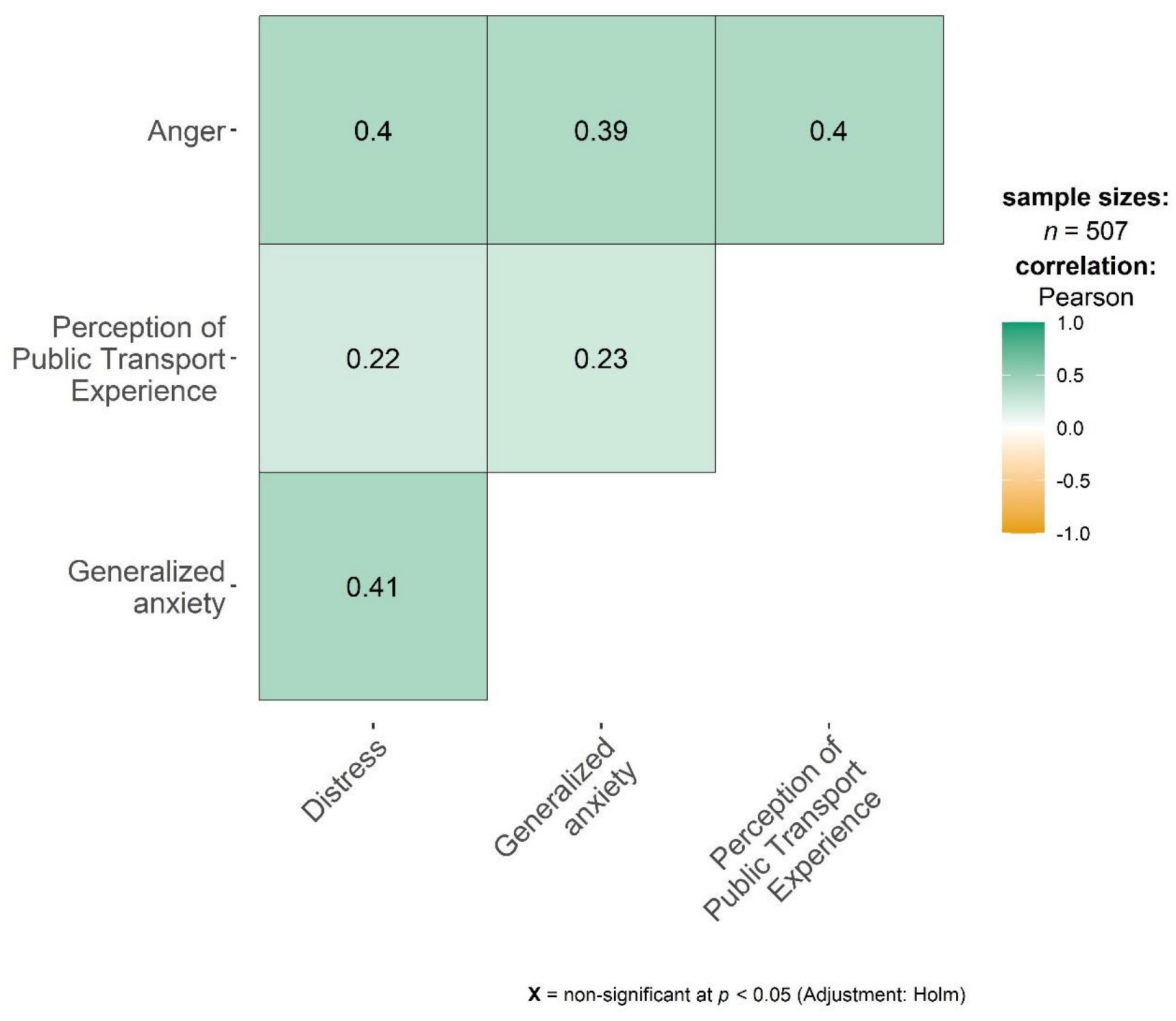
Correlation matrix between perception of public transport experience, distress, generalized anxiety, and anger in adults.

The highest arithmetic mean (M) and standard deviation (SD) were observed for the perception of public transport experience (*M* = 32.15, *SD* = 7.04), whereas the lowest corresponded to generalized anxiety (*M* = 1.25, *SD* = 1.55). Regarding the associations between the psychological variables, the correlation coefficients were statistically significant and ranged in magnitude from small to very large effects, as shown in Figure 1.

### 3.2 Predictive analysis

**Figure 2:**
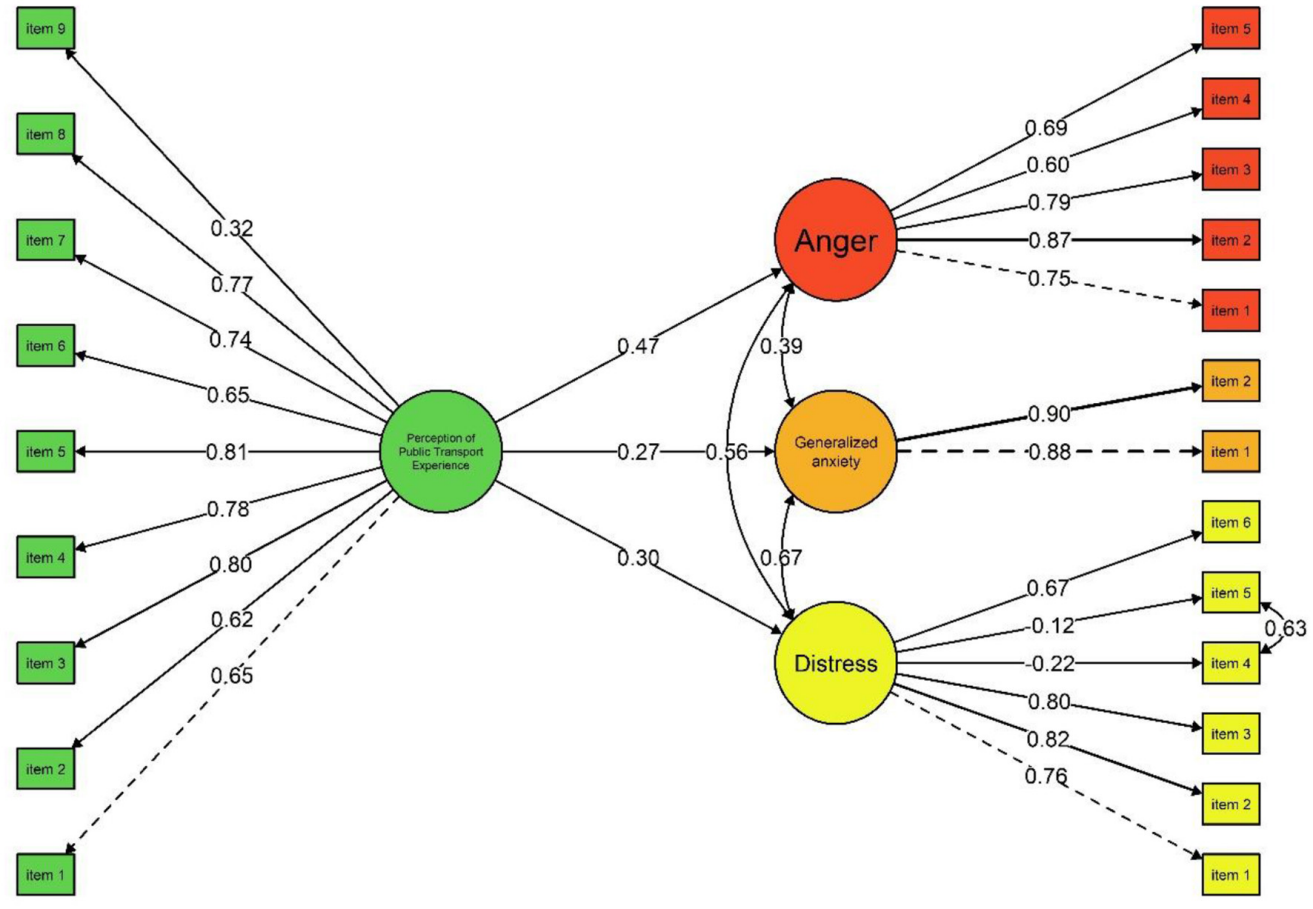
Regression model between perception of public transport experience, distress, generalized anxiety, and anger in adults.

The structural equation modeling analysis achieved acceptable fit after allowing the covariation between one residual error (items 4 and 5 of distress). This covariance was permitted because both items refer to perceptions of uncontrollable and unpredictable events, reflecting highly similar semantic content within the distress construct. The decision was supported by modification indices greater than 10 and by theoretical considerations regarding shared wording and conceptual overlap between these indicators. The model indices were CFI = 0.93, RMSEA = 0.05 [90% CI: 0.049, 0.061], and SRMR = 0.04, the factor loadings, standard errors and *p*-values can be found in Table 2. As shown in Figure 2, the perception of public transport experience was a positive and statistically significant predictor of anger [β = 0.47 (95% CI: 0.375, 0.639), *p* = 0.001], distress [β = 0.30 (95% CI: 0.180, 0.423), *p* = 0.001], and generalized anxiety [β = 0.27 (95% CI: 0.179, 0.389), *p* = 0.001]. In terms of explained variance, the model accounted for 22.4% of the variance in anger (*R*^2^ = 0.224), 8.9% in distress (*R*^2^ = 0.089), and 7.2% in generalized anxiety (*R*^2^ = 0.072). These findings suggest that perception of public transport experience was more strongly associated with anger than with distress or anxiety, indicating a comparatively greater impact on reactive emotional responses than on broader internalizing symptoms.

**Table 2:** Standardized factor loadings, standard errors, and *p*-values of the regression model.

**Items**	**Standardized factor loadings**	**Standard errors**	***p*-values**
**Perception of public transport experience**			
Item 1	0.649	-	-
Item 2	0.617	0.076	0.000
Item 3	0.800	0.073	0.000
Item 4	0.784	0.072	0.000
Item 5	0.809	0.083	0.000
Item 6	0.648	0.083	0.000
Item 7	0.740	0.084	0.000
Item 8	0.769	0.079	0.000
Item 9	0.321	0.084	0.000
**Distress**			
Item 1	0.755	-	-
Item 2	0.817	0.056	0.000
Item 3	0.803	0.070	0.000
Item 4	0.221	0.088	0.001
Item 5	0.125	0.087	0.050
Item 6	0.674	0.072	0.000
**Generalized anxiety**			
Item 1	0.877	-	-
Item 2	0.896	0.052	0.000
**Anger**			
Item 1	0.748	-	-
Item 2	0.871	0.059	0.000
Item 3	0.789	0.077	0.000
Item 4	0.598	0.081	0.000
Item 5	0.693	0.076	0.000

## 4 Discussion

Perception of public transport experience is a daily experience for a large proportion of the adult in urban contexts, particularly in countries such as Peru, where a considerable share of work-, study- and personal-related travel relies on buses, minibuses and other forms of mass transit. These modes of mobility are frequently characterized by adverse conditions such as overcrowding, irregular travel times, continuous exposure to noise and pollution, and a persistent sense of insecurity (Conceição et al., 2023; Kim, 2019; Lan et al., 2020; Wang and Zacharias, 2020). For adults, who often face work demands and family responsibilities, these circumstances become sources of cumulative emotional strain (Carmichael et al., 2024; Tao et al., 2023). In this context, examining the psychological impact of public transport on mental-health-related variables is essential for understanding how seemingly routine daily stressors affect emotional wellbeing. Guided by this premise, the present study aimed to analyse the role of perception of public transport experience as a predictor of anger, distress and generalized anxiety in adults, considering the scarcity of studies employing robust predictive models to examine these links in the Peruvian context.

The results showed positive and statistically significant predictions of anger, distress and generalized anxiety by perception of public transport experience, consistent with in adult populations (Conceição et al., 2023; Hanaee et al., 2025; Lan et al., 2020; Ratering et al., 2024). Notably, the strongest prediction was observed for anger, a finding aligned with the frustration–aggression model. Common commuting experiences—such as unexpected delays, competition for space in overcrowded vehicles or discourteous interactions between passengers and drivers—create ideal conditions for frustration to emerge and, consequently, for intense emotional responses such as anger (Karimi et al., 2022; Ye et al., 2023). These emotional reactions may not remain confined to the commuting context but can spill over into subsequent social interactions, increasing irritability, lowering tolerance, and negatively affecting communication in both workplace and family settings.

This pattern may be particularly pronounced in the Peruvian context, where public transport is characterized by high levels of informality, including the widespread use of “combis,” informal stops, route deviations, and limited regulatory oversight. These features increase unpredictability, perceptions of chaos, and a persistent sense of lack of control during travel. Informal boarding practices, sudden fare negotiations, and aggressive driving behaviors may further heighten passengers' irritation and perceived injustice. Such conditions are especially conducive to anger because they directly threaten individuals' expectations of order, safety, and fairness, which are central antecedents of frustration-based aggression (Karimi et al., 2022). Repeated exposure to these stressors may gradually erode trust and social cohesion among commuters, normalizing hostile or defensive interaction styles during travel and waiting times.

Psychological distress was also significantly predicted by perception of public transport experience. According to environmental stress models, adults who are continually exposed to intense sensory stimuli such as noise, congestion and crowding experience an overload that deteriorates their general emotional state (Chatterjee et al., 2020; Haight et al., 2023; Wang and Zacharias, 2020). Routine perception of public transport experience, particularly when involving prolonged journeys or repeated exposure to unfavorable conditions, contributes to the accumulation of tension and emotional exhaustion (Carmichael et al., 2024; Conceição et al., 2023; Tao et al., 2023). This emotional depletion may reduce individuals' capacity to engage constructively in teamwork, increase interpersonal sensitivity at work, and compromise supportive family interactions.

Generalized anxiety was also significantly predicted, which can be explained through the theory of perceived uncertainty. The possibility of theft, accidents, abrupt route changes or interpersonal conflicts during travel fuels persistent worry among adults—especially those whose commutes are directly tied to job or financial stability (Carmichael et al., 2024; Hanaee et al., 2025; Lan et al., 2020). Chronic commuting-related anxiety may therefore extend into social domains, fostering withdrawal, reduced social participation, and difficulties in sustaining positive relational climates at home and in occupational contexts.

Although the present study did not conduct inferential subgroup comparisons, some descriptive patterns reported in Table 1 may be relevant when considering potential vulnerability. For instance, a considerable proportion of participants reported commuting times exceeding 2 h per day (37.2%), and more than one quarter were not currently employed. Prolonged commuting combined with employment instability may represent contextual conditions associated with increased psychosocial strain (Bai et al., 2021; Carmichael et al., 2024; Evans et al., 2002). These observations are descriptive and non-inferential; however, they suggest that certain subgroups—such as individuals with long daily commutes or precarious employment situations—may experience heightened exposure to transport-related stressors, warranting further stratified research in future studies (Conceição et al., 2023).

Taken together, these findings suggest that while public transport may elicit multiple forms of emotional distress, anger emerges as the most salient response in highly informal and unpredictable transport environments, such as those found in many urban areas of Peru. Overall, these results support the view of public transport as an environmental stressor capable of affecting multiple dimensions of emotional distress and, indirectly, the quality of social relationships and everyday social functioning.

The theoretical implications of these findings are broad. First, the study provides robust evidence that urban mobility should be considered a social determinant of mental health in adults, particularly in contexts where public transport conditions are precarious or unpredictable. The study expands daily stress models by demonstrating that not only work and family environments constitute substantial sources of emotional strain, but also routine activities such as daily commuting (Barros dos Santos and Lima, 2024; Chatterjee et al., 2020; Tao et al., 2023). Moreover, the findings allow for a more specific understanding of the Peruvian case, highlighting how informality, traffic congestion and infrastructural deficiencies intensify the psychological mechanisms involved in transport experiences. In practical terms, the results emphasize the need to integrate mental health into the design and evaluation of mobility policies. Improvements in service frequency, safety, capacity and public transport infrastructure could significantly reduce experiences of anger, stress and anxiety among users (Conceição et al., 2023). At the public-health level, brief psychoeducational interventions within transport systems, such as stress-management guidance or initiatives to promote respectful social interactions, could be implemented. Such strategies may contribute not only to individual wellbeing but also to more positive social climates within transport spaces. From a workplace perspective, promoting flexible schedules or hybrid work models could reduce adults' exposure to stressful transport conditions. The evidence also underscores the importance of planning safer and more human-centered mobility systems, recognizing their real impact on the emotional wellbeing of the Peruvian adults population.

The study has several strengths, including a moderate sample size, which enhances the stability of estimates, and the use of structural equation modeling, which allowed for the simultaneous evaluation of multiple predictive relationships with adequate control of measurement error. However, certain limitations should be noted. The use of a non-probabilistic online sampling strategy limits the generalisability of the findings to the broader adult population of Metropolitan Lima, particularly to individuals without internet access or those who were not reached through social media recruitment. Therefore, the results should be interpreted as reflecting the characteristics of this specific online sample. The cross-sectional design prevents causal inferences, making longitudinal research valuable for assessing the long-term effects of perception of public transport experience. The higher proportion of female participants may introduce bias affecting reported levels of emotional distress, calling for more gender-balanced samples. In addition, reliance on self-report measures may be influenced by social desirability or subjective perception, suggesting the need for complementary physiological or observational measures. Likewise, no comparisons were conducted across sociodemographic subgroups (sex, age group, educational level, employment status, marital status, or commuting time) due to unbalanced categories and the exploratory nature of the SEM, future research should address this limitation through more balanced and stratified sampling designs. Finally, the absence of additional psychological variables, such as coping strategies, resilience or perceived insecurity, restricts a comprehensive understanding of the phenomenon; thus, future studies should consider both risk and protective factors.

## 5 Conclusions

Taken together, the findings indicate that perception of public transport experience is a relevant factor in predicting anger, distress and generalized anxiety in adults. From an environmental psychology perspective, these results underscore how everyday built and social environments shape emotional functioning through continuous person-environment interactions. Daily mobility, far from being a neutral activity, represents a significant component of emotional wellbeing, particularly within the Peruvian context. At a structural level, this evidence highlights the role of transport system quality, reliability, safety, and accessibility as social determinants of mental health. Recognizing its impact supports the development of integrated public policies, urban planning strategies, and transport system improvements aimed at enhancing emotional quality of life, positioning public transport not only as a mobility service but also as a population-level context for mental health promotion and health equity.

## Data Availability

The raw data supporting the conclusions of this article will be made available by the authors, without undue reservation.
